# The use of halloysite clay and carboxyl-functionalised multi-walled carbon nanotubes for recombinant LipL32 antigen delivery enhanced the IgG response

**DOI:** 10.1590/0074-02760140276

**Published:** 2015-02

**Authors:** Daiane D Hartwig, Kátia L Bacelo, Thaís L Oliveira, Rodrigo Schuch, Fabiana K Seixas, Tiago Collares, Oscar Rodrigues, Cláudia P Hartleben, Odir A Dellagostin

**Affiliations:** 1 Programa de Pós-Graduação em Biotecnologia, Núcleo de Biotecnologia, Centro de Desenvolvimento Tecnológico; 2 Departamento de Microbiologia e Parasitologia, Instituto de Biologia, Universidade Federal de Pelotas, Pelotas, RS, Brasil; 3 Departamento de Química, Universidade Federal de Santa Maria, Santa Maria, RS, Brasil

**Keywords:** Leptospira interrogans, nanotubes, LipL32

## Abstract

We studied the feasibility of using halloysite clay nanotubes (HNTs) and carboxyl-functionalised multi-walled carbon nanotubes (COOH-MWCNTs) as antigen carriers to improve immune responses against a recombinant LipL32 protein (rLipL32). Immunisation using the HNTs or COOH-MWCNTs significantly increased the rLipL32-specific IgG antibody titres (p < 0.05) of Golden Syrian hamsters. None of the vaccines tested conferred protection against a challenge using a virulent Leptospira interrogans strain. These results demonstrated that nanotubes can be used as antigen carriers for delivery in hosts and the induction of a humoral immune response against purified leptospiral antigens used in subunit vaccine preparations.

Pathogenic *Leptospira* spp are the agents of leptospirosis, a disease that occurs worldwide, particularly in tropical and subtropical regions (Bharti et al. 2003). Humans and other susceptible animals are infected through contact with the urine of chronic carriers, which are mostly rodents (Faine et al. 1999). In humans, this disease is characterised by fever, renal and hepatic insufficiency and pulmonary manifestations (Adler & de la Peña 2010). The current *Leptospira* vaccines contain whole killed cells of several serovars and induce protective immunity against accidental infection; they are generally reactogenic, require annual booster immunisations and confer serovar-specific immunity (McBride et al. 2005). The latter property is considered the major drawback of these vaccines because there are more than 250 pathogenic serovars of *Leptospira* spp (Bharti et al. 2003).

Outer-membrane proteins, such as the 32-kDa lipoprotein LipL32, are attractive alternatives to leptospiral cellular vaccines due to their antigenic conservation across *Leptospira* serovars (Cullen et al. 2004, Palaniappan et al. 2007 ). Accordingly, there is a need to develop new vaccines that combine a straightforward mode of administration with high efficacy and few side effects. An appealing approach involves the utilisation of effective subunit-based vaccines. However, many new subunit vaccines based on highly purified recombinant proteins are poorly immunogenic and mobilise insufficient immune responses to achieve protective immunity (Zeinali et al. 2009, Foged 2011).

Adjuvants or delivery vehicles are therefore required for vaccine formulations that would enhance, direct and maintain the immune response to vaccine antigens. Some innovative approaches, such as the use of nanovehicles, are currently being employed for this purpose (Pantarotto et al. 2003, Zeinali et al. 2009). Nanotubes are promising because of their propensity to be internalised by a wide variety of cell types *via* several mechanisms (Kam et al. 2005, Kostarelos et al. 2007, Konduru et al. 2009). Here, we present an evaluation of the efficacy of carboxyl-functionalised multi-walled carbon nanotubes (COOH-MWCNTs) and halloysite clay nanotubes (HNTs) in inducing a humoral immune response.

Carbon nanotubes (CNTs) are the most versatile candidate nanostructures for applications in the biomedical, pharmaceutical and biotechnological fields due to their unique physical, chemical and physiological properties (Bianco et al. 2005, Klumpp et al. 2006, Farokhzad & Langer 2009). They are rolled hexagonal carbon networks, of which there are three main types: single-walled CNTs (SWCNTs), double-walled CNTs and multi-walled CNTs (MWCNTs). MWCNTs typically range from 2-100 nm in diameter and 1-50 µm in length. MWCNTs have many advantages; they can be produced on a larger scale, at a lower cost, can be easily functionalised and are biocompatible (Gao et al. 2006). Furthermore, well-functionalised, dispersed CNTs do not appear to have inherent toxicity (Dumortier et al. 2006) and can carry a large number of peptide ligands. There is also evidence that CNTs can produce immune responses when covalently linked to highly immunogenic peptide sequences (Pantarotto et al. 2003).

HNTs are composed of an economically viable clay material that can be mined from deposits as a raw mineral. Halloysite is a 1:1 aluminosilicate clay mineral with the empirical formula Al_2_Si_2_O_5_(OH)_4_. The predominant form of HNTs is a hollow tubular structure that is 500-1,000 nm in length and 15-100 nm in inner diameter, depending on the deposit (Lvov et al. 2008). The inner lumens of HNTs can be loaded with a range of materials, such as macromolecules and proteins. Biocompatibility is one of the main prerequisites for the safe usage of halloysite for the delivery of biologically active substances (Vergaro et al. 2010). Thus, the objective of the present study was to evaluate MWCNTs and HNTs with respect to LipL32 recombinant protein (rLipL32) antigen delivery in vivo and to determine their capacity to enhance the IgG antibody response against this antigen relative to a leptospirosis control.

*Leptospira interrogans* serovar Copenhageni strain Fiocruz L1-130, originally isolated from a patient with severe leptospirosis (Ko et al. 1999), was the source of the genomic DNA used in the present study. The cloning, expression and purification of the rLipL32 was performed as previously described (Seixas et al. 2007). For protein purification, the cell pellets were harvested, suspended in purification buffer (200 mM NaH_2_PO_4_, 0.5 M NaCl and 5 mM imidazole, pH 8.0) and incubated on an orbital shaker at 60 rpm for 18 h at room temperature. Purification was performed using immobilised metal ion-affinity chromatography using Ni^2+^ Sepharose HisTrap columns (GE Healthcare, USA). The purified protein was dialysed against phosphate-buffered saline (PBS) and the concentration was determined using a BCA Protein Assay Kit (Pierce, USA).

MWCNT and HNTs were obtained from Sigma^(r)^ (USA) and the MWCNTs were carboxylated at Department of Chemistry, Federal University of Santa Maria, state of Rio Grande do Sul, Brazil, according to a previously described method (Stefani et al. 2011). The oxidation and characterisation of COOH-MWCNTs was conducted using X-ray photoelectron spectroscopy and Raman spectroscopy.

The immunogenicity of rLipL32 associated with the HNTs, COOH-MWCNTs or Alhydrogel (InvivoGen, USA) was determined using groups of six female Golden Syrian hamsters of four-six-weeks of age. The negative control groups were injected with only the nanotubes or with Alhydrogel. Thus, the vaccine groups were as follows: rLipL32-COOH-MWCNTs, rLipL32-HNTs, rLipL32-Alhydrogel, PBS-COOH-MWCNTs, PBS-HNTs and PBS-Alhydrogel. The rLipL32 dose administered was 50 µg mixed with HNTs (at 75 µg·mL^-1)^, COOH-MWCNTs (at 15 µg·mL^-1)^ or Alhydrogel (at 15%). The vaccine preparations were formulated by mixing rLipL32 with the HNTs, COOH-MWCNTs or Alhydrogel, in the concentrations described above and allowing them to adsorb for 24 h prior to performing the immunisations. The quantities of rLipL32 protein in samples of rLipL32-COOH-MWCNT, rLipL32-HNT, rLipL32-Alhydrogel, PBS-COOH-MWCNT, PBS-HNT and PBS-Alhydrogel vaccines was determined using sodium dodecyl sulfate polyacrylamide gel electrophoresis (SDS-PAGE), with visualisation through Coomassie-blue staining and quantification using a NanoVue(tm) Plus Spectrophotometer (GE Healthcare).

Booster doses were administered 14 days after the first immunisation. Sera were collected from the retro-orbital plexus at zero, 14 and 28 days post-immunisation (DPI). Twenty-eight days after the first immunisation, the hamsters were challenged with an intraperitoneal inoculum of 1.3 × 10^3^ leptospires of *L. interrogans* serovar Copenhageni strain Fiocruz L1-130. The hamsters were monitored daily and they were euthanised and considered dead when the clinical signs of terminal disease reached a moribund level.

The IgG immune response induced by the vaccine preparations was determined using an ELISA against the rLipL32 antigen, using methodology previously described (Hartwig et al. 2011). Briefly, microtitre wells were coated with 200 ng of rLipL32, washed and blocked using blocking buffer (PBS pH 7.4, 0.05% Tween 20 and 5% non-fat dried milk). Hamster sera diluted 1:50 was added and IgG antibodies were detected using a rabbit anti-Golden Syrian hamster IgG antibody conjugated to peroxidase (Rockland Immunochemicals Inc, USA) at a dilution of 1:6,000. The reaction was visualised using *o*-phenylenediamine dihydrochloride (Sigma-Aldrich) and hydrogen peroxide. The reaction was stopped by adding 0.1 M sulphuric acid and the absorbance at 492 nm was determined using a Multiskan MCC/340 ELISA plate reader (Titertek Instruments, USA). The mean values were calculated from sera samples assayed in triplicate.

An analysis of variance was used to identify significant differences between the assay results. The Student *t* test and the Tukey test were employed to determine significant differences in the results of the serological assays. Differences were considered significant at a p value of *< *0.05.

We tested the hypothesis that using nanotubes in subunit vaccine preparations would enhance the delivery of the recombinant antigenic peptide and the induction of peptide-specific immune responses in vivo. Functionalised CNTs have been used to successfully deliver and present antigenic peptides to the immune system (Pantarotto et al. 2003, Villa et al. 2011). In those studies, the authors demonstrated that CNTs effectively presented peptides to the humoral immune system; however, the effects of the CNT-based delivery on the cellular immune response were not clear. In our study, the immunised hamsters had a significantly greater anti-rLipL32 IgG response compared with that of the negative control groups (p < 0.05) (Figure). In the hamsters that were immunised using rLipL32-COOH-MWCNTs or rLipL32-HNTs, the IgG response observed at 14 DPI was significant. At 28 DPI, the immune response of the hamsters that had been immunised using those vaccine preparations and the hamsters that had been immunised using rLipL32-Alhydrogel was increased. The Tukey multiple comparisons test demonstrated that, at 14 DPI, the rLipL32-HNTs had induced an IgG immune response that was significantly different from the response induced by rLipL32-Alhydrogel, but was not different from the response induced by the rLipL32-COOH-MWCNTs. Furthermore, the antibody response induced by the rLipL32-COOH-MWCNTs did not differ from that induced by rLipL32 mixed with Alhydrogel. At 28 DPI, the animals that received rLipL32 administered with HNTs or COOH-MWCNTs had a significantly greater IgG response compared with that of the rLipL32-Alhydrogel group. The immunisations using PBS-COOH-MWCNTs, PBS-HNTs and the PBS-Alhydrogel negative control did not induce detectable levels of IgG antibodies. All of the immunised hamsters died at nine-12 days post challenge, indicating that the vaccine preparations had not induced protective immunity.

**Figure:**
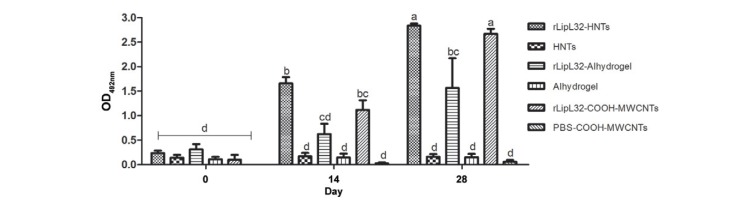
IgG immune response stimulated by LipL32 recombinant protein (rLipL32) associated with halloysite clay nanotubes (HNTs), carboxyl-functionalised carbon nanotubes (COOH-MWCNTs) or Alhydrogel adjuvant determined by ELISA. Values are presented as means ± standard error of the means of two independent experiments. Letters represent a difference between groups (p < 0.05). Samples were analysed in triplicate. OD: optical density; PBS: phosphate-buffered saline.

Previous studies suggested that the protective immune response against leptospirosis is antibody dependent (Palaniappan et al. 2006, Chang et al. 2007, Silva et al. 2007). However, high agglutinating antibody titres against lipopolysaccharide in the serum of vaccinated cattle were not sufficient for protection (Bolin et al. 1989). Some authors have suggested that cell-mediated immunity is also important in the protection of humans and other animals (Brown et al. 2003). Therefore, new carriers or adjuvant molecules must be evaluated with respect to their ability to induce a broad immune response, particularly those materials that can efficiently deliver antigens into dedicated antigen-presenting cells, such as dendritic cells.

Nanotubes have tremendous potential in a number of different applications, including delivering antigens and stimulating immunity. CNTs represent a class of emerging materials that can penetrate cells without causing cellular death (Pantarotto et al. 2003). Recently, it was demonstrated that functionalised CNTs conjugated to peptides generated an immune response in the absence of potential cytotoxic effects (Zeinali et al. 2009). The authors stated that COOH-SWCNT doses ranging from 5-15 µg·mL^-1^ did not affect cell viability. We tested COOH-MWCNTs under several concentrations in Chinese hamster ovary cells and observed no significant in vitro cytotoxic activity using the (2-methoxy-4-nitro-5-sulphophenyl)-2H-tetrazolium-5-carboxanilide assay (data not shown). We chose to use 15 µg·mL^-1^ of the COOH-MWCNTs in our experiments. HNTs inhibited cell growth in a concentration-dependent manner and cell viability was preserved when the halloysite dose was as high as 75 µg·mL^-1^ (Vergaro et al. 2010). In contrast to HNTs, CNTs are difficult to use in vaccine preparations due to their lack of solubility in many solvents. However, their carbon atoms present an excellent platform for chemical functionalisation and carboxyl groups can be added to CNTs to improve their dispersion in water (Zhao et al. 2010). The MWCNTs used in this study were functionalised using carboxyl groups and thus were easily solubilised in water for use in vaccine preparations.

The quantities of rLipL32 that adsorbed to HNTs, COOH-MWCNTs or Alhydrogel in the vaccine preparations was determined using SDS-PAGE (data not shown) and spectrophotometry. The results of this assay demonstrated that only 12% (6 µg) and 24% (12 µg) of rLipL32 protein had adsorbed to COOH-MWCNTs and HNTs, respectively, and we believe that this may have occurred through a physical interaction, although we did not investigate this hypothesis. In comparison, Alhydrogel adsorbed more than 80% (40 µg) of the protein. In our immunogenicity assays using an rLipL32 subunit vaccine, we included a group in which Alhydrogel was used as an adjuvant. Alhydrogel is regularly used in commercial animal vaccines and is approved for use in human and animal vaccines (Petrovsky & Aguilar 2004). We used this adjuvant as a standard in our evaluations because it has been shown to enhance the humoral immune response against rLipL32 (Seixas et al. 2007). In conclusion, the data presented here demonstrated that HNTs and COOH-MWCNTs induced a strong IgG immune response against a purified leptospiral antigen; the response was more robust than that induced by Alhydrogel, suggesting that these nanotubes can be considered new carriers for the delivery of biomolecules, such as proteins and peptides.
